# Medical and surgical management of pancreatic fluid accumulations in dogs: A retrospective study of 15 cases

**DOI:** 10.1111/jvim.16411

**Published:** 2022-03-23

**Authors:** Charles T. Talbot, Ring Cheung, Emma J. Holmes, Simon D. Cook

**Affiliations:** ^1^ Department of Veterinary Clinical Science and Services, Royal Veterinary College University of London Hertfordshire United Kingdom; ^2^ Royal Veterinary College University of London London United Kingdom; ^3^ Department of Pathobiology and Population Sciences, Royal Veterinary College University of London Hertfordshire United Kingdom

**Keywords:** cytology, necrosis, pancreatitis, pseudocyst, ultrasound

## Abstract

**Background:**

Limited data exist on the diagnosis and successful medical management of suspected pancreatic abscessation, and the appropriate terminology of this condition.

**Hypothesis/Objectives:**

To describe the diagnosis and management of pancreatic fluid accumulations in dogs where pancreatic fluid cytology results were available, to describe those medically and surgically managed at the same institution, and reconsider the terminology describing acute pancreatitis with pancreatic fluid accumulation.

**Animals:**

Fifteen dogs treated for suspected pancreatic abscessation at a university teaching hospital between January 2010 and March 2020.

**Methods:**

Retrospective descriptive study.

**Results:**

Ultrasonographic findings described pancreatic fluid accumulations as hypoechoic in 10/15 and anechoic in 2/15 cases, ranging between 1.6 and 7 cm in diameter (median, 3.5 cm). No complications were documented after ultrasound guided fine‐needle aspiration. Cytologically, all samples revealed a predominantly neutrophilic inflammation. 11/15 samples yielded a negative culture (9/11 received antimicrobials before sampling) and in 4 cases culture was positive. 7/15 were initially managed surgically including all 4 infected cases. 4/7 surgically managed cases were discharged, including 2 infected cases. The remaining 3/7 surgically managed cases were euthanized due poor quality of life. 8/15 cases were managed medically; 7/8 were discharged, 1 died. 3/7 then represented, and underwent successful surgical intervention after recrudescence of clinical signs, and all were discharged. The remaining 4 medically managed cases did not require further therapeutic intervention, with no clinical deterioration on reassessment.

**Conclusions and Clinical Importance:**

Medical management is a viable treatment option for some dogs with pancreatic fluid accumulation, or as a prequel to surgical management. Subclassifications of pancreatic fluid accumulations using diagnostic findings could enable more tailored management approaches and accurate prognostication.

**Abbreviations**
cPL: canine pancreatic lipase
TNCC: total nucleated cell count
TP: total protein

## 1 INTRODUCTION

Pancreatic fluid accumulations (or abscesses) are infrequently reported within the veterinary literature, described as collections of necrotic, purulent material, with or without infection, and as suspected sequelae to an acute inflammatory pancreatopathy.^1^, ^2^, ^3^, ^4^ The precise definitions and underlying pathophysiology of these processes have not been determined in veterinary medicine; however, the first human consensus classification system of these inflammatory pancreatic fluid accumulations was established in 1992.^5^ A 2012 revision proposed 4 subtypes of pancreatic fluid collections: acute peripancreatic fluid collections (APFC), acute necrotic collections (ANC), pseudocysts, and walled‐off necrosis (WON).^6^ Acute necrotic collections are on a spectrum with WON, when an organized wall is yet to be formed.

The term “abscess” is traditionally defined as a circumscribed collection of purulent material, but is often used to encompass these pancreatic processes indiscriminately and has been abandoned in the human literature as it is deemed too inaccurate and ambiguous.^6^ The medical and veterinary communities have used the term “pancreatic phlegmon” to describe sterile or septic pancreatic lesions encompassing edematous to necrotizing pancreatitis; however, this term has also been discouraged on similar grounds.^3^, ^5^

Human guidelines for the management of pancreatic fluid accumulations mandate the differentiation between a septic or sterile process, which tend to require surgical or medical management, respectively.^5^ However, less invasive, staged approaches involving percutaneous drainage catheter placement have been described and documented to reduce new‐onset multiple organ failure when compared to open necrosectomy, without increased risk of reintervention, even in infected cases.^7^, ^8^ The medical community consensus is that pancreatic fluid accumulations do not resolve spontaneously, and that drainage is considered necessary.^9^ The veterinary field has infrequently reported resolution of these pancreatic processes through conservative management.^2^, ^10^

Pancreatic fluid accumulations reported in the veterinary literature are infrequently and inconsistently defined, with no clear treatment guidelines. Surgical findings or gross descriptors such as the mucopurulent nature of fluid are often used to define them.^1^, ^2^, ^11^, ^12^ These accumulations are often considered to be infected without cytological evidence,^2^ but infection rates in dogs appear lower than in people, with between 15% and 25% yielding a positive culture compared to 40% to 70%, respectively.^12^, ^13^, ^14^

Surgical approaches in dogs are often advocated despite high case fatality rates of 33% to 86%,^1^, ^2^, ^11^, ^12^ especially when a septic process is identified.^15^ Surgical techniques reported include debridement, excision, omentalization, and peritoneal drainage by open or closed technique.^1^, ^2^, ^11^, ^13^

Successful medical management of an infected pancreatic abscess in a cat with concurrent diabetes mellitus has been reported.^16^ The cat was treated supportively with antibiotics and percutaneous drainage on 2 occasions, 2 weeks apart, with a complete resolution of the abscess. There is no literature specifically describing the successful medical management of pancreatic abscessation in dogs. The objectives of this study were to describe the diagnostic testing, management, and outcomes among dogs with pancreatic fluid accumulations, and to discuss current disease classification shortcomings.

## 2 MATERIALS AND METHODS

### 2.1 Case selection

Cases were retrospectively identified between January 2010 and March 2020 by evaluating computer records at the Queen Mother Hospital for Animals, Royal Veterinary College for all dogs with clinicopathological findings consistent with pancreatitis based on measurement of canine pancreatic specific lipase or ultrasonographic findings, together with suspected pancreatic abscessation. Pancreatic abscessation was defined as ultrasonographic evidence of a fluid filled lesion and cases were only included where cytological examination of the fluid was available. No ethical approval was required for this study.

### 2.2 Medical records review

The search terms “pancreatic abscessation” and “pancreatic abscess” were used to search the computerized records of the Queen Mother Hospital for Animals for dogs between January 2010 and March 2020. Inclusion criteria were the presence of a pancreatic fluid accumulation from which fine needle aspirate cytology was available, and the presence of an active pancreatopathy with a working diagnosis of “pancreatic abscessation.” Details recorded included signalment, reason for referral, comorbidities, duration of clinical signs before presentation, physical examination findings, hematology and biochemistry analyses, fine needle aspirate cytology, microbiology, and treatment details. The ultrasonographic findings including size and location of the lesions were recorded. Treatment was determined by the primary clinician, but details over the decision‐making process for medical versus surgical management were recorded when available. Medical versus surgical case classification was determined based on the management from initial presentation until discharge. Follow‐up was sought and included where available through the evaluation of medical and telecommunication records. The information obtained included the presence or absence of ongoing clinical signs, physical examination findings, and if applicable, repeated ultrasonographic findings. Exclusion criteria were incomplete medical records, and the presence of peripancreatic fluid accumulation rather than pancreatic. All ultrasonographic findings and cytological interpretations were interpreted by a boarded radiologist, or boarded clinical pathologist, respectively.

### 2.3 Statistical methods

Descriptive statistics were calculated as appropriate using commercially available software (Microsoft Excel; Version 15.35; Microsoft Corporation, One Microsoft Way, Redmond, Washington). Due to small sample sizes, further analyses were not performed.

## 3 RESULTS

Initial searches yielded 27 cases, which were reviewed by 2 authors for inclusion. Eleven cases were excluded due to insufficient cytological data, and 1 case was excluded due to insufficient ultrasonographic findings. Fifteen dogs remained in the study.

### 3.1 Signalment

Of the 15 dogs included, 9 were male, of which 7 were neutered. Six were female, all of which were neutered. Median age was 95 months (range, 24‐151 months). Breeds represented included Labrador retriever (n = 2), Yorkshire terrier (n = 2), and 1 of each of the following breeds: jack russell terrier, Irish red setter, cross breed, golden retriever, cavapoo, labradoodle, beagle, cocker spaniel, English bull terrier, English springer spaniel, and Chihuahua cross. Concurrent diseases included diabetes mellitus (n = 2), idiopathic epilepsy (n = 2), high grade perianal adenocarcinoma (n = 1), and diabetic ketoacidosis (n = 1).

### 3.2 Initial investigations

**FIGURE 1:**
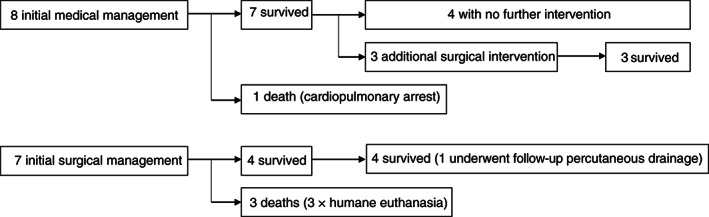
Management and survival of medically and surgically managed cases with pancreatic fluid accumulation

Physical examination findings on presentation are summarized in Table 1. Results of hematology and biochemistry analyses are summarized in Table 2. Eight dogs were managed medically, and 7 surgically. Figure 1 summarises the treatment and outcome of all 15 cases.

**TABLE 1:** Physical examination findings in medically and surgically managed cases

	Medical	
	Median	Range
Temperature (°C)	38.6	38.3 ‐ 39.5
Heart rate (bpm)	120	80 ‐ 148
Respiratory rate (brpm)	36	12 ‐ 60

	Surgical	
	Median	Range
Temperature (°C)	38.7	38.4 ‐ 40.1
Heart rate (bpm)	108	76 ‐ 200
Respiratory rate (brpm)	25	20 ‐ 32

**TABLE 2:** Hematology (Advia 2120i Hematology Analyzer, Siemens) and biochemical (ILab 500, Werfen) results between medically and surgically managed cases

	Medical		Surgical		
	Median	Range	Median	Range	Reference range
Neutrophils (10^9^/L)	16.4	4.6 ‐ 46.8	12.8	5.2 ‐ 31.6	3 ‐ 11.5
Platelets (10^9^/L)	228.5	24 ‐ 377	296	76.4 ‐ 352	150 ‐ 900
Packed cell volume (%)	40.5	32 ‐ 46	36	11 ‐ 44	37 ‐ 55
Albumin (g/L)	25.6	21.2 ‐ 32.4	23.7	16 ‐ 34.6	28 ‐ 39
cPL (μg/L)	964.5	144 ‐ 2000	1000	137 ‐ 2000	<200
Glucose (mmol/L)	6.4	3.4 ‐ 10.1	21	7.2 ‐ 34	4.7 ‐ 7.3

### 3.3 Medical cases

#### 3.3.1 Canine pancreatic lipase

Canine pancreas‐specific lipase (cPL) activity was quantified in 6/8 dogs, with a median of 964.5 μg/L (range, 144‐2000 μg/L; reference range, <200 μg/L). One dog had a cPL of less than 200 μg/L; the remaining 5 being above 400 μg/L. All samples were sent to an external clinical pathology laboratory (IDEXX Laboratories, Inc, United Kingdom). In 1 case, only a qualitative cPL SNAP test was performed and reported abnormal, indicative of a positive, above reference range reading. In 1 case, no cPL testing was performed; biochemical testing identified an amylase of 2752 (reference range, 176‐1245 U/L) and lipase of 1658 (reference range, 72‐1115 U/L).

#### 3.3.2 Ultrasound

All medically managed dogs had a full abdominal ultrasound examination performed or overseen by a board‐certified diagnostic imaging specialist. Findings included a hypoechoic pancreas in 5 cases, an enlarged or thickened pancreas in 5 cases, and increased echogenicity of the surrounding mesentery in 6 cases. Pancreatic fluid accumulation contents were subjectively described as hypoechoic in 5 cases, anechoic in 1 and were uncharacterized in 2 cases, with a median lesion diameter of 3.1 cm (range, 1.6‐7 cm). Six dogs had peritoneal fluid, of which 3 were minimal in volume, 2 were moderate, and 1 moderate to large.

#### 3.3.3 Cytology—pancreatic

Fine needle aspirates of pancreatic fluid accumulations revealed predominantly neutrophilic inflammation in all cases with evidence of calcification (n = 6) and necrosis (n = 4). Intact pancreatic epithelial cells were identified in only 2 cases, in low numbers and without abnormalities. No bacteria were identified in any case.

#### 3.3.4 Bacteriology

Culture of pancreatic abscess fluid was performed in 6/8 cases, all were negative.

#### 3.3.5 Cytology—peritoneal

Peritoneal effusions were identified and sampled in 6 cases and classified as neutrophilic exudates. Median total nucleated cell count (TNCC) was 39.0 × 10^9^/L (range, 3.8‐77.2 × 10^9^/L) (n = 6). Median total protein (TP) was 35.0 g/L (range, 25.9‐55 g/L) (n = 5). Canine pancreatic lipase was not measured in any effusion. No bacteria were identified in any case.

#### 3.3.6 Management

The median duration of clinical signs before presentation was 4 days (range, 2‐19 days). Justification for medical management included a positive response to initial medical treatment (n = 5), financial constraints (n = 1), perceived surgical risk due to systemic instability and severity of clinical picture (n = 1), or client preference despite the recommendation of surgical management (n = 1). Treatment included IV administration of fluid therapy, gastroprotectants, antiemetics, antinausea medication, opioid analgesia, and nutritional support (Table S1). Antimicrobials were administered to 4 of the medically managed dogs, all of which were initiated before sampling of the pancreatic lesions. Amoxicillin and clavulanic acid was used in 2 and cefuroxime in 1 dog. The antibiotic used before referral was not disclosed in 1 case. In surviving dogs that received antibiotics, these were administered for a median of 3 days (range, 3‐6 days). Where voluntary nutrition was deemed insufficient, it was supplemented by nasogastric, or nasoesophageal means in 2 dogs (Table S1).

#### 3.3.7 Outcome

Of the 8 dogs initially treated medically, 7 survived to discharge, with a median hospitalization duration of 5.5 days (range, 4‐20 days), and 1 sustained cardiopulmonary arrest after a 6‐day progressive, systemic inflammatory response syndrome (SIRS) clinical picture and subsequent development of respiratory distress. A post mortem of this dog was not performed. Of the 7 dogs that were discharged, 4 had individual follow‐up assessments on day 7, 23, 42 or 58 after discharge, respectively, documenting no recurrence of clinical signs or deterioration, and were clinically well before being lost to follow up. Follow‐up ultrasound was performed in 1 of these, at 20 days after the initial diagnostic imaging. Findings included evidence of an ongoing pancreatopathy (hypoechoic pancreas and hyperechoic surrounding mesenteric fat) but with reduced pancreatic thickness, less irregular margins, and complete resolution of the pancreatic fluid accumulation. The 3 remaining cases were readmitted for surgical intervention (including lavage, debridement, and omentalization, with or without active peritoneal drainage), due to recurrent clinical signs of lethargy, vomiting and inappetence after 1, 8, and 17 days, and were subsequently successfully discharged again.

### 3.4 Surgical cases

#### 3.4.1 Canine pancreatic lipase

Canine pancreatic lipase activity was quantified in 5/7 dogs with a median concentration of 982.3 μg/L (range, 137‐2000 μg/L; reference range, <200 μg/L). Only 1 sample had a cPL of less than 200 μg/L; the remaining 4 being above 400 μg/L.

#### 3.4.2 Ultrasound

All surgically managed dogs had a full abdominal ultrasound examination performed or overseen by a board certified diagnostic imaging specialist. Findings included a hypoechoic pancreas in 5 cases, an enlarged or thickened pancreas in 4 cases and increased echogenicity of the surrounding mesentery in 6 cases. Pancreatic fluid accumulation contents were subjectively described as hypoechoic in 5 cases, anechoic in 1 case, and uncharacterized in 1 case, with a median diameter of 3.5 cm (range, 2.5‐7 cm). Six cases had peritoneal effusion, 5 of which were minimal in volume, and in 1 the volume was not described.

#### 3.4.3 Cytology—pancreatic

Fine needle aspirates of pancreatic fluid accumulations revealed predominantly neutrophilic inflammation and calcification in all cases with evidence of necrosis in the majority of cases (n = 6). Intracellular rod bacteria were identified in 2 cases. Intact pancreatic epithelial cells were identified in 1 case with slight dysplastic features, secondary to inflammation.

#### 3.4.4 Bacteriology

Culture of pancreatic fluid was performed in 5/7 of cases, of which 4 were positive. The bacteria cultured included *Escherichia coli* (n = 2), *Staphlococcus pseudointermedius* (n = 1), or a combination of both *Staphlococcus pseudointermedius* and *E. coli* (n = 1). The remaining culture was negative. Of the 4 samples which were positive, cytology identified rod bacteria in 1 case. The other sample in which intracellular bacteria were identified cytologically was not cultured.

#### 3.4.5 Cytology—peritoneal

Peritoneal effusions were identified in 4 cases and classified as neutrophilic exudates, with a median TNCC of 52.2 × 10^9^/L (range, 40.5‐84.2 × 10^9^/L) and a TP of 30.6 g/L (range, 21‐34.5 g/L). No intracellular bacteria were identified on cytological evaluation; however, 1 cytologically negative sample suspicious for a septic process yielded a positive bacterial culture. Canine pancreatic lipase was not measured in any effusion.

#### 3.4.6 Management

The median duration of clinical signs before presentation was 16 days (range, 4‐27 days). The median duration of time between referral presentation and surgical management was 3 days (range, 0‐7 days). Justification for surgical management included further investigation through biopsy of the pancreatic abscess (n = 2), the ultrasonographic appearance (n = 1), worsening clinical signs (n = 1), suspicion of a septic process (n = 1), a positive bacterial culture of the pancreatic fluid accumulation (n = 1), or septic peritonitis secondary to a previous enterotomy (n = 1; Table S1). Surgical techniques included debridement, lavage, omentalization, with or without placement of an indwelling peritoneal drain.

Six of 7 dogs received antibiotics, all of which were administered before sampling of the pancreatic fluid accumulation. The antibiotics used in 4 dogs were 1 or a combination of: amoxicillin and clavulanic acid (n = 3), enrofloxacin (n = 2), metronidazole (n = 1) and marbofloxacin (n = 1). The antibiotics received by the remaining 2 dogs before referral were not disclosed. In those dogs that received antibiotics, these were administered for a median of 5.5 days (range, 5‐28 days). In the 1 surviving dog that received antibiotics, these were administered for a total of 28 days. Where voluntary nutrition was deemed insufficient, it was supplemented via esophageal tube (n = 2), jejunostomy tube (n = 1), or nasogastric tube (n = 1) for a median of 4.5 days (range, 3‐6 days) before voluntary intake was sustained (Table S1).

#### 3.4.7 Outcome

Of the 7 dogs initially managed surgically, 4 survived to discharge after a median of 9 days of hospitalization (range, 4‐13 days), and 3 were euthanized due to a deterioration in quality of life and a perceived poor prognosis. Of those euthanized, 2 had positive bacterial cultures from pancreatic fluid. Follow‐up was achieved for 3/4 successful cases up to 10, 29, and 54 days after discharge, at which stage all were clinically well, with no documented clinical signs, or signs of deterioration. Follow‐up ultrasound examinations were performed on 2 of these dogs. In 1 dog this was performed 9 days after the initial ultrasound examination; there was ongoing evidence of pancreatic fluid accumulation, and the lesion was percutaneously drained. Cytology of the repeated percutaneous drainage confirmed septic neutrophilic inflammation. Culture and sensitivity results documented *E. coli* with sensitivity to the previously prescribed enrofloxacin, which was continued for a total of 4 weeks. Ultrasound of the same site 29 days later documented complete resolution of the fluid accumulation. In the other dog, imaged 54 days after the initial ultrasound examination, there was no evidence of fluid accumulation.

## 4 DISCUSSION

Diagnostic sampling of pancreatic fluid accumulations is safe and allows for both cytological and microbiological assessment to differentiate between a sterile and a septic process. The decision to pursue either medical or surgical management was determined by clinician preference, with all infected cases undergoing surgical management. Medical management appears to be a viable and safe option for certain cases, even where surgical management is ultimately deemed necessary. This is in line with staged approaches in people.^7^

Fine needle aspiration is the diagnostic procedure of choice in people for identifying infective processes in pancreatic necrotic collections.^17^ Fine needle aspirates were performed under ultrasound guidance in all 15 dogs, with no complications identified during or after the sampling process. Similar sampling techniques have been described in a previous veterinary study, with no complications reported via leakage of pancreatic pseudocyst contents into the abdominal cavity.^4^ Cytology revealed a markedly inflammatory picture with evidence of necrosis in the majority of samples. These findings parallel the descriptions of acute necrotic collections and WON characterized in people, some with associated infection.^6^

Bacterial culture was positive in 4/15 dogs, with cytological examination identifying 2 of these. Previous literature reports positive culture frequencies of 15% and 25%.^12^, ^13^ Of those which were identified as infected, all 4 received antibiotics before sampling. Of those in which a culture was performed across both groups (n = 11), 7 had antibiotics administered before sampling. It is difficult to discern if the use of antibiotics resulted in a low yield of aerobic or anaerobic cultures. Dogs with a positive bacterial culture appeared more likely to be considered surgical, in line with current literature in people.^5^, ^18^

There are no accepted diagnostic criteria in veterinary medicine that accurately describe or differentiate acute pancreatic fluid collections, and the term “abscess” has been avoided where possible in this study. Future attempts should be made to classify acute pancreatic fluid collections, for example as per the guidelines in people. Fine needle aspirate sampling is expected to improve these classifications and could carry therapeutic potential where drainage is performed; reducing an ongoing inflammatory or infectious process in line with minimally invasive approaches in people.^7^, ^8^, ^16^, ^19^ This was achieved in 1 of the surgically managed dogs in which a residual pancreatic fluid accumulation was identified on reassessment. This fluid was drained percutaneously, with a complete resolution on further reassessment 29 days later.

Current recommendation for treatment of infected fluid collections as a result of necrotizing pancreatitis in people include minimally invasive approaches such as percutaneous or endoscopic drainage.^7^, ^8^, ^18^, ^20^ Given the acceptance and success of these minimally invasive techniques, exploration of similar practices in veterinary medicine could hold promise. The high case fatality rates associated with surgery in both veterinary medicine and in people have directed recent recommendations toward conservative management as an alternative or prequel to more invasive procedures.^4^, ^21^

In addition to the 4 medically managed dogs that did not require surgery, 1 of the surgically managed dogs subsequently represented and had a pancreatic fluid accumulation drained ultrasonographically before making a full recovery. When considered in conjunction with the 3 cases that failed medical management yet underwent successful surgical management, conservative approaches appear to be safe practice, even if not curative. A period of medical management before determining a need for surgery appears to be safe and parallels recommendations in people; the cases that were managed medically initially and discharged either continued this trajectory of improvement, or underwent successful surgical intervention due to a resumption of clinical signs.

In both the medical and surgically managed groups, a large proportion of animals received antibiotics. In people, differentiating those cases with septic pancreatic fluid accumulations guides the use of antibiotics.^22^ Antibiotic usage is only justified in those cases where an infected necrosis has been diagnosed either by cytology or culture.^23^ The veterinary field is yet to establish similar guidelines specific to pancreatic fluid accumulations, however, until such time antibiotics should be reserved for when infection is documented by either cytology or culture.^24^ Similarly, the use of gastric protectants in these cases ought to be reserved for those with genuine gastrointestinal mucosal compromise such as gastroduodenal ulceration, or gastroesophageal reflux disease.^25^

There are no accepted management recommendations for pancreatic necrotic collections in veterinary medicine, only a tendency for surgical management.^12^, ^26^ This course of treatment is associated with high case fatality rates, with survival ranging between 33% and 86%.^1^, ^2^, ^11^, ^12^ Of the 4 dogs in this study with infection of the fluid accumulation, all underwent surgical management, and 2 survived to discharge. The identification of infection was the primary justification for surgery in 1 dog, with unclear motives in the remaining 3. It is possible that an infective process is associated with a worse outcome, but further studies would be required to support this. Recommendations in people propose surgical intervention if minimally invasive approaches such as percutaneous drainage have failed to resolve a septic process; this could be open surgery, minimally invasive surgery, endoscopic surgery, or a combination of these.^18^

The recent abandonment of the term “pancreatic abscessation” in the human literature and recognition of acute peripancreatic fluid collections (APFC), acute necrotic collections (ANC), pseudocysts, and walled‐off necrosis (WON) subtypes has allowed more accurate classification in diagnostic, therapeutic and prognostic research.^6^ APFC develop within the early phase of pancreatitis, and do not have a well‐defined encapsulating wall. They are typically sterile, and resolve without intervention. A pancreatic pseudocyst is a fluid collection which contains no solid necrotic material, in the peripancreatic tissues, and is surrounded by a well‐defined wall. ANC develop within the first 4 weeks of pancreatitis and contain variable amounts of fluid and necrotic tissue. Advanced imaging techniques such as contrast enhanced computed tomography, or abdominal ultrasonography, identify variable amounts of solid necrotic material and fluid, and contents which might appear loculated. Acute necrotic collections are delineated from APFCs as they contain necrotic material, and arise from sites of necrotizing pancreatitis. WON contains necrotic tissue, which is contained within a contrast enhancing fibrous wall of reactive tissue. They are typically seen after maturation, which could take more than 4 weeks after the onset of necrotizing pancreatitis.^6^ Based on the classifications in people, it is likely that the cases described in this study include ANCs or WON, with and without infection. These subtypes are categorized by diagnostic imaging findings, specifically ultrasonography, and by fine needle aspirate cytology. With the increasing availability of advanced imaging in veterinary medicine, a similar standardization could be possible, allowing for a more objective evaluation, and development of appropriate treatment plans for each subtype. A recent case study described WON in a dog as per the human medicine reclassification system, using ultrasonography and computed tomography.^27^ The dog underwent a partial pancreatectomy, and the findings were confirmed histopathologically. Three months following partial pancreatectomy, ultrasonography was unremarkable with a complete resolution of clinical signs.

The major limitations of this study were its retrospective nature and the differing case management strategies which could even have changed over time. Low case numbers also inhibited more detailed comparisons and statistical analyses, and follow‐up was limited to the short‐term.

## 5 CONCLUSION

The present study identified that ultrasound guided fine needle aspirate sampling of pancreatic fluid accumulations appears to be safe, and that medical management appears to be a viable treatment option in some dogs, with those failing undergoing successful surgical procedures. The intrapancreatic lesions in these cases likely represent ANCs or WON based on both ultrasonographic and cytological findings, with some exhibiting concurrent infective processes. The utilization of an improved veterinary classification system along with further studies, as seen in the human literature, would improve the ambiguity of pancreatic fluid collection definitions and could allow for more accurate treatment guidelines and prognostication.

## CONFLICT OF INTEREST DECLARATION

Authors declare no conflict of interest.

## OFF‐LABEL ANTIMICROBIAL DECLARATION

Authors declare no off‐label use of antimicrobials.

## INSTITUTIONAL ANIMAL CARE AND USE COMMITTEE (IACUC) OR OTHER APPROVAL DECLARATION

Authors declare no IACUC or other approval was needed.

## HUMAN ETHICS APPROVAL DECLARATION

Authors declare human ethics approval was not needed for this study.

## Supporting information


**Table S1** Medical and surgical management of pancreatic fluid accumulation
