# Supplementary material for: Medical and surgical management of pancreatic fluid accumulations in dogs: A retrospective study of 15 cases
Source: J Vet Intern Med. 2022 Mar 23;36(3):919–26. doi: 10.1111/jvim.16411 (PMC9151450; doi:10.1111/jvim.16411)
Supplement: Supplementary file 1 — Table S1 Medical and surgical management of pancreatic fluid accumulation [file JVIM-36-919-s001.pdf]

Supplementary Table 1: Medical and surgical management of pancreatic fluid accumulation

Signalment					Management			Survival	Treatment							
**#**	**Age (months)**	**Sex**	**Breed**	**Comorbidities**	**Initial Treatment**	**Additional Treatment**	**Surgical Approach**	**Discharge (including additional treatment)**	**Antibiotics**	**Culture**	**Intravenous Fluid Therapy**	**Analgesia**	**Antiemetic**	**Gastric Protectants**	**Routes of nutritional delivery**	**Diet**
1	146	MN	Labrador	Perianal adenocarcinoma	Medical	None	-	Yes	None	Negative	N/A	N/A	N/A	N/A	N/A	N/A
2	137	FN	Jack russel terrier	Diabetes mellitus	Medical	None	-	Yes	None	Negative	CSL	Buprenorphine (0.01 – 0.02mg/kg IV q8hr)	Maropitant (1mg/kg IV q24h)	Omeprazole 1mg/kg IV q12h)	N/A	N/A
3	60	FN	Irish red setter		Medical	Surgery	Debridement, lavage, omentalization, Jackson-Pratt drain	Yes	Amoxicillin and clavulanic acid	Negative	N/A	N/A	N/A	N/A	N/A	N/A
4	44	MN	Cross breed	DIC, thrombocytopenia	Medical	None	-	Yes	Cefuroxime	Negative	CSL	Tramadol (2mg/kg PO q8h)	Maropitant (1mg/kg IV q24h)	Omeprazole (1mg/kg IV q12h)	Nasoesophageal and oral	N/A
5	103	ME	Golden retriever	Epilepsy	Medical	None	-	No	Amoxicillin and clavulanic acid	Negative	CSL	Methadone (0.2mg/kg IV q4h)	Maropitant (1mg/kg IV q24h)	Omeprazole (1mg/kg IV q12h)	Nasogastric and oral	Enteral care
6	104	FN	Cavapoo		Medical	None	-	Yes	None	Negative	CSL	Methadone (0.1 – 0.2mg/kg IV q4h)	Maropitant (1mg/kg IV q24h)	Omeprazole (1mg/kg IV q12h)	Oral	Royal Canin GI low fat
7	95	MN	Labradoodle	Idiopathic epilepsy	Medical	Surgery	Debridement, lavage, omentalization, Jackson-Pratt drain	Yes	N/A	Negative	CSL	Methadone (0.2mg/kg IV q4h), buprenorphine 0.01 – 0.02mg/kg IV q8h)	Maropitant (1mg/kg IV q24h), ondansetron (0.5mg/kg IV q8h)	Omeprazole (1mg/kg PO q12h)	Oral	Chicken
8	118	FN	Beagle		Medical	Surgery	Lavage, omentalization	Yes	None	Negative	CSL	Methadone (0.2 – 0.3mg/kg IV q4h), paracetamol (10mg/kg IV q12h)	Maropitant (1mg/kg IV q24h)	None	Oral	Royal Canin GI low fat
9	151	MN	Cocker spaniel		Surgical	None	Debridement, lavage, omentalization	Yes	N/A	Staphylococcus pseudintermedius	CSL	Buprenorphine (0.01mg/kg IV q8h), tramadol (2mg/kg PO TID)	Maropitant (1mg/kg IV q24h)	Omeprazole (1mg/kg PO q12h)	Esophageal and oral	N/A
10	60	MN	English bull terrier		Surgical	None	N/A	No	Marbofloxacin, amoxicillin and clavulanic acid	Negative	CSL	N/A	N/A	N/A	N/A	N/A
11	24	FN	Labrador		Surgical	None	Debridement, lavage, omentalization	Yes	N/A	Negative	N/A	N/A	N/A	N/A	Jejunostomy and oral	N/A
12	96	MN	Yorkshire terrier		Surgical	None	N/A	No	Enrofloxacin, amoxicillin and clavulanic acid	Staphylococcus pseudintermedius & Escherichia Coli	N/A	N/A	N/A	N/A	Esophageal and oral	N/A
13	30	MN	Jack russell terrier	Septic peritonitis	Surgical	Percutaneous drainage	Debridement, lavage, omentalization, Jackson-Pratt drain	Yes	Enrofloxacin	Negative	N/A	N/A	N/A	N/A	N/A	N/A
14	65	FN	Chihuahua X	Diabetes mellitus	Surgical	None	Debridement, lavage, omentalization	Yes	None	Escherichia Coli	CSL	Methadone (0.1 – 0.2mg/kg IV q4h)	None	None	Oral	Chicken, Royal Canin GI low fat
15	70	MN	Yorkshire terrier	Diabetic ketoacidosis	Surgical	None	Debridement, lavage, Jackson-Pratt drain	No	Metronidazole, amoxicillin and clavulanic acid		CSL	Fentanyl (1 – 5 mcg/kg/hr IV CRI), ketamine (0.1 – 0.5 mcg/kg/hr IV CRI), Methadone (0.2mg/kg IV q4h)	Maropitant (1mg/kg IV q24h), medetomidine (0.5mg/kg.hr)	Omeprazole (1mg/kg IV q12h), sucralfate (75mg/kg PO q8h)	Nasogastric and oral	Royal Canin GI low fat

N/A, not available; IV, intravenous; PO, per os; CRI, constant rate infusion; CSL, compound sodium lactate
